# Validity and reliability of the Swedish version of the Children’s Sleep Habits Questionnaire (CSHQ-SWE)

**DOI:** 10.1186/s12887-024-04859-z

**Published:** 2024-05-31

**Authors:** Ingrid Larsson, Petra Svedberg, Jens M. Nygren, Julia S. Malmborg

**Affiliations:** School of Health and Welfare, Halmstad University, Box 823, Halmstad, SE-301 18 Sweden

**Keywords:** ADHD, Children’s sleep habits questionnaire, CSHQ, Sleep, Sleep actigraphy, Reliability, Validity

## Abstract

**Background:**

To translate and culturally adapt the Children’s Sleep Habits Questionnaire (CSHQ) to a Swedish version, CSHQ-SWE, and to assess its validity and reliability for use with children with attention deficit hyperactivity disorder (ADHD).

**Methods:**

A total of 84 children with ADHD (51 boys and 33 girls; 6–12 years) and parents (7 men and 77 women; 28–51 years) were included in the study. CSHQ was translated and culturally adapted to Swedish, and assessed for concurrent validity with sleep actigraphy (analyzed by Kendall’s Tau) and for reliability by internal consistency (analyzed by McDonald’s Omega H). Face and content validity was evaluated by parents (*n* = 4) and healthcare professionals (*n* = 6) qualitatively (comprehensiveness, relevance, and comprehensibility assessed by interviews and analyzed by thematic analysis) and quantitatively (analyzed by content validity ratio and content validity index for 33 items and four non-scored inquiries).

**Results:**

Parent-reported sleep problems (CSHQ-SWE total score) were moderately correlated with less “Sleep Efficiency” (Tau = −0.305; p < 0.001) measured by sleep actigraphy. Parent-reported problems with “Sleep Onset Delay” was moderately correlated with measured time for ”Sleep Onset Latency” (Tau = 0.433; *p* < 0.001). Parent-reported problems with “Night Wakings” were weakly correlated with measured time for “Wake After Sleep Onset” (Tau = 0.282; *p* < 0.001). Parents estimation of “Total daily sleep duration” was moderately correlated with measured “Total Sleep Time” (Tau = 0.386; *p* < 0.001). Five of the seven subscales reached an acceptable level for internal consistency (McDonald’s Omega H > 0.700). Comprehensiveness, relevance, and comprehensibility of CSHQ-SWE were satisfactory overall. Content validity ratio was 0.80 to 1.00 for six items, 0.00 to 0.60 for 22 items, and < 0.00 for nine items. Content validity index was 0.22.

**Conclusions:**

CSHQ-SWE demonstrated acceptable concurrent validity with objectively measured sleep and internal consistency, whereas the overall results of face and content validity assessment varied. The instrument needs to be further evaluated regarding construct validity, responsiveness, test-retest reliability, and its generalization to other populations.

**Supplementary Information:**

The online version contains supplementary material available at 10.1186/s12887-024-04859-z.

## Background

Sleep is essential for health and well-being, and the recommended daily amount of sleep for children aged 6–12 years to achieve health benefits, is 9–12 h [1]. Various dimensions of sleep-related issues impact the overall sleep experience, including sleep problems and sleep disorders [2]. For instance, sleep disorders, such as insomnia and sleep-related movement disorders, represent specific clinical conditions. Meanwhile, night wakings emerge as a sleep problem, which may manifest as a symptom of various sleep disorders. Sleep assessment instruments are important for capturing these various dimensions of sleep [3], for following trends and changes in sleep habits on a population level [4], for evaluating outcomes of sleep interventions [2, 5], and for evaluating treatment effects for patients in clinical settings [2]. Sleep may also be assessed in relation to overall health [6].

The Children’s Sleep Habits Questionnaire (CSHQ) is a widely used sleep assessment instrument capturing various dimensions of sleep problems. It was originally published in English [3], and translations and validations have been made in several languages with satisfactory psychometric properties [7–12]. Several CSHQ validation studies have focused on the general population and excluded children with neurodevelopmental disorders [3, 7, 11, 12]. Despite this, the CSHQ has been used to assess sleep in children with neurodevelopmental disorders [6, 13–16], and this prompts the need for the adaptation and validation of CSHQ for this patient population. It is important in such efforts to include the children with neurodevelopmental disorders in the instrument development, since they seem to be specifically affected by sleep problems [6, 17, 18] compared to the general population [15, 19]. Parreira et al. [19] have evaluated the validity of the CSHQ for children with attention deficit hyperactivity disorder (ADHD) from Portugal, finding adequate psychometric properties, which suggests that CSHQ may be suitable to adapt. Additionally, Hansen et al. [16] conducted a study evaluating the internal consistency of the CSHQ for children with ADHD from Norway.

In summary, there is a lack of questionnaires in Swedish that assess children’s sleep problems in general, and in children with neurodevelopmental disorders in particular. The aim of this study was to translate and culturally adapt the CSHQ to a Swedish context and to assess the validity and reliability of the CSHQ-SWE in children with ADHD.

## Methods

### Design and setting

This study is part of a randomized, placebo-controlled crossover trial with a sleep intervention with weighted blankets for children with uncomplicated ADHD and sleep problems [20]. Children and parents (or legal guardians) were recruited in couples to the research project. The present study had an instrument development design based on the CSHQ and was performed in two phases. Phase 1 involved translation and cultural adaptation of CSHQ to the Swedish language and culture, CSHQ-SWE, and assessment of concurrent validity and reliability, and phase 2 involved the assessment of face and content validity of CSHQ-SWE.

### Participants and recruitment

In 2019–2022, children referred to an ADHD unit at a Child and Adolescent Mental Health Service (CAMHS) in southern Sweden were invited to participate in the research project. The research project included children who met specific criteria: a diagnosis of ADHD, presence of sleep problems, stable medication, absence of melatonin medication, and no prior experience with weighted blankets. Children diagnosed with uncomplicated ADHD (without significant comorbidities or social burden) falling within the Diagnostic and Statistical Manual of Mental Disorders, Fifth Edition (DSM-5) spectrum, the Inattentive, Hyperactive, or Combined subtypes, without significant comorbidities warranting primary concern for pharmacological or psychosocial interventions, were included in the study. A senior child and adolescent psychiatrist diagnosed the children when attending the ADHD unit according to DSM-5. Screening for sleep problems was conducted by a healthcare professional using three questions from the Children’s Sleep Habits Questionnaire [3]: Question 2 (“Child falls asleep within 20 minutes after going to bed”, scoring criteria 0–4 days per week), question 9 (“Child sleeps too little”, scoring criteria 2–7 days per week), and question 25 (“Child awakes more than once during the night”, scoring criteria 2–7 days per week). Besides fulfilling the scoring criteria for at least one of the three questions, parents also needed to acknowledge the sleep issue as a problem for their child. Thus, the psychometric properties of the questionnaire in our study are assessed towards sleep problems and not clinical sleep disorders.

A total of 643 children were referred to the ADHD unit during the recruitment period. Of these children, 154 were eligible for participation. After applying inclusion criteria, including an age limit of 6–12 years for the current study, 63 children were ineligible or unwilling to participate, leaving 91 children, each accompanied by one of their parents (or a legal guardian), in the final sample.

### The Children’s Sleep Habits Questionnaire (CSHQ)

The CSHQ targets parents and is designed to subjectively evaluate sleep habits and sleep problems in children. It comprises 33 questions and is validated for use with children aged 4–10 years [3], but has also been validated and frequently used with children up to 12 years of age [6, 21, 22]. For questions 1–31, parents are required to estimate the frequency of the events named in the questionnaire during the past week, or during a typical week, using the following response options: “Usually” (occurring 5 times or more during a week, assigned 3 points), “Sometimes” (occurring 2 to 4 times during a week, assigned 2 points), or “Rarely” (occurring never or once a week, assigned 1 point). Questions 1, 2, 3, 10, 11, and 26 are scored in reverse order. Questions 32 and 33 focus on assessing sleepiness during various activities and are answered using the following response options: “Not sleepy” (assigned 1 point)”, “Very sleepy” (assigned 2 points), or “Falls asleep” (assigned 3 points). The total score ranges between 33 and 99. A higher total score indicates a greater likelihood of sleep problems. Additionally, sleep problems can be further analyzed across eight subscales: “Bedtime Resistance” (questions 1, 3, 4, 5, 6, and 8), “Sleep Onset Delay” (question 2), “Sleep Duration” (questions 9, 10, and 11), “Sleep Anxiety” (questions 5, 7, 8, and 21), “Night Wakings” (questions 16, 24, and 25), “Parasomnias” (questions 12, 13, 14, 15, 17, 22, and 23), “Sleep Disordered Breathing” (questions 18, 19, and 20), and “Daytime Sleepiness” (questions 26, 27, 28, 29, 30, 31, 32, and 33). Moreover, the questionnaire also includes non-scored inquiries related to (a) bedtime, (b) total daily sleep duration combining nighttime sleep and naps (in hours and minutes), (c) nighttime awakenings (estimated as minutes per awakening), and (d) the time of morning awakening [3]. CSHQ items, subscales, and non-scored inquiries are presented in an additional file [see Additional file 1].

### The Swedish version of the Children’s Sleep Habits Questionnaire (CSHQ-SWE)

#### Translation and cultural adaptation

With permission from the original author [3], the CSHQ was translated from English to Swedish in several steps [23, 24]. Step 1: Two independent researchers (IL and KA), native speakers of Swedish, translated the English original into two Swedish versions. Step 2: The two translations were synthesized to form one shared version. Step 3: A professional (non-informed) native English translator back-translated the shared version into English. Step 4: A multidisciplinary team with various professions, methodological expertise, and specific competencies (IL – registered nurse, KA – physiotherapist, HJ – pediatric physician specialized in psychiatry, PS – specialist psychiatric nurse, JMN – health science researcher, JSM – exercise physiologist) reviewed, together with the English translator, the Swedish shared version and the English back-translated version, and a back-and-forth process took place until consensus was reached regarding wording, semantics, idiomatics, culture, and concepts. The multidisciplinary team had previous experience in instrument development, validation processes, and questionnaire studies. This resulted in CSHQ-SWE, which was first piloted in a subgroup of eight parents and then distributed to parents at the baseline of the research project.

#### Concurrent validity

Concurrent validity was assessed by analyzing correlations between the CSHQ-SWE and sleep actigraphy. Actigraphy, frequently employed to investigate sleep patterns among children [25] has undergone validation in pediatric populations, including children with ADHD [25, 26]. The use of actigraphy was chosen for our study due to its practicality and ability to monitor sleep patterns in a home environment, minimizing disruption and enhancing data collection reliability.

At baseline, sleep was measured objectively by a wrist-worn actigraph (Motionware 1.2.47 Camntech). Parents received both written and verbal instructions regarding the actigraph, specifying that it should be worn on the non-dominant wrist at least 1–2 h before bedtime until after waking up and getting up in the morning. The parent or child was also instructed to press a marker button when it was time to sleep or turn off the lights. The actigraph was worn for a minimum of 3 nights [27] and a maximum of 7 nights. Each night was analyzed separately and then presented as an average for the period. Data was analyzed with a medium sensitivity setting [25, 28] and an epoch length of 30 s. The software algorithm suggested bedtime and wake-up times, but all data were manually scored and checked by three researchers (ML, KA, and JSM) to ensure concordance with digital sleep diaries. These diaries consisted of daily text messages informing the data analysis process regarding bedtime and wake-up times [5, 20, 25]. The marker was preferred when substantial disparities were noted between the “lights out” time recorded by the marker and the daily text messages. Likewise, actigraphy data was prioritized when significant differences were observed between the wake-up time recorded by the actigraph and the information from text messages. In instances of uncertainty, scorers engaged in discussions to reach a consensus.

The sleep actigraphy measurements were “Sleep Onset Latency” (SOL), “Wake After Sleep Onset” (WASO), “Total Sleep Time” (TST), and “Sleep Efficiency” (SE). SOL describes the minutes between turning off the lights and falling asleep. WASO describes minutes of wakefulness after falling asleep. TST describes minutes of sleep between sleep onset and morning awakening. SE describes the ratio between TST and duration of time in bed (lights off to morning awakening) and is multiplied by 100 to achieve a percentage value [5]. Concurrent validity was assessed by analyzing correlations between the CSHQ-SWE (total score, subscales, and the non-scored inquiry “Total daily sleep duration”) and the sleep actigraphy measurements (SOL, WASO, TST, and SE).

#### Reliability

Reliability was assessed at baseline by internal consistency, reflecting agreement between items included in the same subscale. McDonald’s Omega H was used to assess internal consistency for all CSHQ-SWE subscales, except for “Sleep Onset Delay”, a single-item subscale. McDonald’s Omega H was selected because the analysis permits various factor loadings on items within the same construct [29].

#### Face and content validity

Face and content validity of the CSHQ-SWE were further explored after the sleep intervention period had been completed. This was performed with individual interviews with four parents (women) of children with ADHD. Face and content validity were also assessed by healthcare professionals in a focus group interview (five women) and in an individual interview (one man). The healthcare professionals were a pediatric physician specialized in psychiatry, two specialist psychiatric nurses, a nurse assistant, and two psychologists from a child and adolescent mental health service. They had an average of 22 years as professionals (range 5–42 years) and of 19 years within a psychiatry clinic (range 4–40 years). The interview guide consisted of questions concerning the questionnaire’s comprehensiveness, relevance, and comprehensibility. The health professionals were also asked about the applicability of the questionnaire. Two experienced researchers performed the interviews (IL and JSM), and the interviews were transcribed verbatim. The total time for all the interviews was four hours and three minutes.

Content validity for each question was also rated by the parents and the healthcare professionals, where 0 = Not necessary, 1 = Useful, and 3 = Essential. Content validity ratio (CVR) was calculated for each item by the equation: CVR = (n_e_ – N / 2) / (N / 2), where n_e_ is number of participants that rated the item as essential, and N is the total number of participants. Content validity index (CVI) was calculated using the mean CVR for all items [30, 31].

### Data analysis

The scoring of CSHQ-SWE was performed in accordance with the manual [3]. The total score and subscales for CSHQ-SWE were computed for all the complete CSHQ-SWE questionnaires. For questionnaires with less than 20% of missing items, the mean value from the specific subscale was imputed [8]. No official guidelines for imputation were found for questions 5 and 8 that appear in two subscales. To avoid overestimation of sleep problems, the missing values on these questions were substituted with the lowest mean value of the subscales “Bedtime Resistance” or “Sleep Anxiety”. If more than 20% of items were missing, the questionnaire was excluded from analyses [8].

Statistical analyses for assessing concurrent validity were performed with IBM SPSS Statistics software v.28 (IBM Corp., Armonk, NY, USA), and for assessing reliability with JASP (version 0.18.1). Statistical significance was set to *p* < 0.05. Age was presented as mean and standard deviation (SD). For assessment of concurrent validity, normal distribution of data was first analyzed with the Shapiro Wilks test. Data was presented descriptively with median and interquartile range (IQR) as the data was not normally distributed. Differences between boys and girls were analyzed with Mann-Whitney U test (effect size r) before correlation analyses were performed. Kendall’s Tau was used for correlation analyses as the dataset was small, the data were not normally distributed, and there were several tied ranks for CSHQ-subscales. Correlation coefficient and bootstrapped 95% bias corrected and accelerated confidence interval (BCa CI) were presented [32]. A correlation coefficient of 0.10–0.29 was considered a weak correlation, 0.30–0.49 a moderate correlation, and 0.50–1.0 a strong correlation [33]. For assessment of reliability, McDonald’s Omega H was presented with 95% confidence interval (CI). Values of 0.700 or higher were considered acceptable [29].

The analysis of face and content validity interviews was inspired by thematic analysis [34] including the concepts of comprehensiveness, relevance, and comprehensibility of CSHQ-SWE. Transcripts were read several times, and suggestions, remarks, and thoughts from participants were thematized with regard to similarities and differences. Interviews were evaluated by IL, PS, and JSM.

CVR may range between − 1 (no panel member scoring item as essential) and 1 (all panel members scoring item as essential) which means that a value of 0.00 indicates agreement between 50% of panel members on an item being essential [35]. For a panel member size of 10, a CVR of 0.62 indicates the minimum acceptable value [31].

## Results

Of the 91 children and parent couples eligible for this study in the research project, three had more than 20% missing items on CSHQ, two were treated with melatonin, and two did not wear the actigraph. This resulted in the exclusion of seven couples from the analysis. The remaining 84 children were 51 boys (60.7%) and 33 girls (39.3%) with a mean age of 9.0 ± SD 1.9 years (range 6–12 years). The 84 parents were seven men (8.3%) and 77 women (91.7%) with a mean age of 38.6 ± SD 5.1 years (range 28–51 years). Imputation of values for CSHQ-SWE was made in 0.4% of all questions (12 / (33 × 84)) with imputation of two questions for four individuals and one question for four individuals. The total score for CSHQ-SWE ranged between 37 and 71 points in the sample. There were no differences between boys and girls regarding CSHQ-SWE scores and sleep actigraphy results (Table 1), and all were included in subsequent correlation analyses.

**Table 1:** Mann-Whitney U tests for CSHQ-SWE and sleep actigraphy measurements

	Descriptives median (IQR Q1–Q3)			Mann-Whitney U test boys vs. girls			
	All, *n* = 84	Boys, *n* = 51	Girls, *n* = 33	U	z	Asymp. sig. (2-tailed)	Effect size *r*
**CSHQ-SWE**							
Total score (range 33–99)*	52.00 (48.00–57.75)	53.00 (48.00–58.00)	52.00 (48.00–56.50)	797.00	−0.41	0.683	−0.04
Bedtime Resistance (range 6–18)*	8.00 (7.00–12.00)	9.00 (7.00–12.00)	8.00 (7.00–12.50)	812.50	−0.27	0.789	−0.03
Sleep Onset Delay (range 1–3)*	2.00 (2.00–3.00)	2.00 (2.00–3.00)	2.00 (2.00–3.00)	806.00	−0.35	0.725	−0.04
Sleep Duration (range 3–9)*	5.00 (4.00–7.00)	5.00 (4.00–7.00)	6.00 (4.00–6.50)	840.50	−0.01	0.993	0.00
Sleep Anxiety (range 4–12)*	6.50 (4.00–8.00)	7.00 (5.00–8.00)	6.00 (4.00–8.50)	743.00	−0.92	0.359	−0.10
Night Wakings (range 3–9)*	4.50 (3.00–6.00)	5.00 (3.00–7.00)	4.00 (3.00–5.00)	692.50	−1.40	0.163	−0.15
Parasomnias (range 7–21)*	9.00 (8.00–10.00)	9.00 (8.00–10.00)	8.00 (7.00–10.50)	737.00	−0.97	0.330	−0.11
Sleep Disordered Breathing (range 3–9)*	3.00 (3.00–3.00)	3.00 (3.00–3.00)	3.00 (3.00–3.50)	835.50	−0.07	0.941	−0.01
Daytime Sleepiness (range 8–24)*	16.00 (13.00–18.00)	15.00 (12.00–18.00)	16.00 (14.00–18.00)	731.00	−1.02	0.310	−0.11
Total daily sleep duration (min)**	540.00 (480.00–586.75)	540.00 (480.00–547.00)	540.00 (495.00–600.00)	783.00	−0.54	0.587	−0.06
**Sleep actigraphy measurements**							
SOL (min)	29.07 (16.43–48.07)	30.43 (17.29–49.14)	22.71 (12.86–40.86)	723.50	−1.08	0.280	−0.12
WASO (min)	39.00 (30.89–48.82)	39.43 (32.67–53.29)	37.40 (29.00–47.21)	703.00	−1.27	0.205	−0.14
TST (min)	494.36 (460.36–533.46)	491.43 (460.57–529.29)	509.86 (459.57–535.50)	801.50	−0.37	0.714	−0.04
SE (%)	87.01 (83.84–90.05)	86.34 (83.70–88.86)	88.43 (84.07–90.84)	682.50	−1.46	0.145	−0.16

*Total possible ranges for the CSHQ-SWE.

** Includes nighttime sleep and naps

Higher values reflect more sleep problems for all variables, except for Total daily sleep duration, TST, and SE, where lower values reflect more sleep problems

IQR, Interquartile Range; Min, Minutes; Q, Quartile; SE, Sleep Efficiency; SOL, Sleep Onset Latency; TST, Total Sleep Time; WASO, Wake After Sleep Onset

### Concurrent validity

Results from the correlation analyses with Kendall’s Tau showed that the CSHQ-SWE total score, subscales (except subscale “Sleep Disordered Breathing”), and “Total daily sleep duration” item were significantly correlated in various degrees with one or several sleep actigraphy measures (Table 2). A moderate correlation was found with SE for CSHQ-SWE total score, where more parent-reported sleep problems were associated with less sleep efficiency (Tau = −0.305; 95% BCa CI −0.425 to −0.169; *p* < 0.001). Parent-reported problems adhering to CSHQ-SWE subscale “Sleep Onset Delay” was moderately correlated with measured time for SOL (Tau = 0.433; 95% BCa CI 0.295 to 0.547; *p* < 0.001). Parent-reported problems adhering to CSHQ-SWE subscale “Night Wakings” were weakly correlated with measured nightly awake time reflected by WASO (Tau = 0.282; 95% BCa CI 0.096 to 0.452; *p* < 0.001). The parents’ estimation of children’s “Total daily sleep duration” was moderately correlated with measured TST (Tau = 0.386; 95% BCa CI 0.223 to 0.534; *p* < 0.001).

Additional weak correlations were found between CSHQ-SWE subscale “Sleep Duration” and SOL (Tau = 0.255; 95% BCa CI 0.109 to 0.407; *p* = 0.001), CSHQ-SWE subscale “Sleep Anxiety” and WASO (Tau = 0.265; 95% BCa CI 0.104 to 0.423; *p* < 0.001), and CSHQ-SWE subscale “Daytime Sleepiness” and SOL (Tau = 0.244; 95% BCa CI 0.125 to 0.352; *p* = 0.002).

**Table 2:** Kendall’s Tau for CSHQ-SWE and sleep actigraphy measurements

	Sleep actigraphy measurements							
	SOL*		WASO*		TST**		SE**	
	Kendall’s Tau (95% BCa CI)	*p*-value	Kendall’s Tau (95% BCa CI)	*p*-value	Kendall’s Tau (95% BCa CI)	*p*-value	Kendall’s Tau (95% BCa CI)	*p*-value
**CSHQ-SWE**								
Total score*	0.282 (0.134 to 0.410)	< 0.001	0.091 (−0.040 to 0.229)	0.230	−0.030 (−0.163 to 0.101)	0.693	−0.305 (−0.425 to −0.169)	< 0.001
Bedtime Resistance*	0.049 (−0.097 to 0.198)	0.530	0.192 (0.051 to 0.329)	0.014	0.128 (−0.033 to 0.279)	0.100	−0.090 (−0.241 to 0.054)	0.248
Sleep Onset Delay*	0.433 (0.295 to 0.547)	< 0.001	−0.069 (−0.232 to 0.116)	0.422	−0.091 (−0.282 to 0.105	0.286	−0.279 (−0.426 to −0.127)	0.001
Sleep Duration*	0.255 (0.109 to 0.407)	0.001	−0.110 (−0.257 to 0.062)	0.171	−0.156 (−0.311 to 0.007)	0.052	−0.228 (−0.390 to −0.076)	0.004
Sleep Anxiety*	−0.048 (−0.224 to 0.121)	0.543	0.265 (0.104 to 0.423)	< 0.001	0.231 (0.080 to 0.382)	0.003	−0.058 (−0.205 to 0.092)	0.465
Night Wakings*	−0.023 (−0.180 to 0.146)	0.773	0.282 (0.096 to 0.452)	< 0.001	0.055 (−0.109 to 0.204)	0.494	−0.138 (−0.298 to 0.019)	0.085
Parasomnias*	0.141 (−0.009 to 0.302)	0.078	0.105 (−0.052 to 0.251)	0.188	0.006 (−0.153 to 0.159)	0.940	−0.192 (−0.328 to −0.062)	0.016
Sleep Disordered Breathing*	−0.037 (−0.254 to 0.173)	0.682	0.078 (−0.095 to 0.256)	0.389	0.031 (−0.150 to 0.206)	0.729	−0.059 (−0.233 to 0.129)	0.512
Daytime Sleepiness*	0.244 (0.125 to 0.352)	0.002	−0.198 (−0.343 to −0.055)	0.010	−0.186 (−0.329 to −0.040)	0.016	−0.170 (−0.305 to −0.015)	0.027
Total daily sleep duration**	−0.247 (−0.390 to −0.093)	0.002	0.138 (−0.025 to 0.299)	0.080	0.386 (0.223 to 0.534)	< 0.001	0.208 (0.054 to 0.344)	0.008

*Higher values reflect more sleep problems

**Lower values reflect more sleep problems

Bootstrap results are based on 1000 samples

BCa, bias corrected and accelerated; CI, confidence interval; SE, Sleep Efficiency; SOL, Sleep Onset Latency; TST, Total Sleep Time; WASO, Wake After Sleep Onset

### Reliability

McDonald’s Omega H was acceptable (≥ 0.700) for the CSHQ-SWE subscales “Bedtime Resistance” (Omega H = 0.827; 95% CI 0.770–0.883), “Sleep Duration” (Omega H = 0.798; 95% CI 0.725–0.872), “Sleep Anxiety” (Omega H = 0.729; 95% CI 0.637–0.820), “Night Wakings” (Omega H = 0.811; 95% CI 0.694–0.889), and “Daytime Sleepiness” (Omega H = 0.768; 95% CI 0.694–0.842). McDonald’s Omega H for the CSHQ-SWE subscale “Parasomnias” was below the acceptable level (Omega H = 0.573; 95% CI 0.429–0.718) and the subscale ”Sleep Disordered Breathing” was not possible to analyze due to the poor distribution of answers to the questions included in the subscale (Table 3).

**Table 3:** McDonald’s Omega H for CSHQ-SWE subscales

	McDonald’s Omega H (95% CI)
**CSHQ-SWE subscales (no. of questions)**	
Bedtime Resistance (6)	0.827 (0.770–0.883)
Sleep Onset Delay (1)	N.A.
Sleep Duration (3)	0.798 (0.725–0.872)
Sleep Anxiety (4)	0.729 (0.637–0.820)
Night Wakings (3)	0.811 (0.694–0.889)
Parasomnias (7)	0.573 (0.429–0.718)
Sleep Disordered Breathing (3)	*
Daytime Sleepiness (8)	0.768 (0.694–0.842)

* Not possible to analyze. Sample size = 84. CI, confidence interval; N.A., not applicable; No, number

### Face and content validity

#### Comprehensiveness, relevance, and comprehensibility

For comprehensiveness, the CSHQ-SWE was experienced as being well adapted to investigating children’s sleep by both parents and healthcare professionals. Several external factors, such as medication, gaming, physical activity, food habits, intake of beverages with sugar and/or caffeine, screen time, sedentary time, school stress, room temperature, and noise disturbances, were highlighted by parents and healthcare professionals as factors impacting on children’s sleep. The multidisciplinary team acknowledged that several of the above-mentioned factors are important aspects of sleep, but that no questions needed to be added to CSHQ-SWE since these factors are not within the scope of the questionnaire. Healthcare professionals also wanted to know more about the duration of sleep problems, and perceived sleep quality. They suggested that question 31 (“Child seems tired”) could be divided into “Child does not seem well-rested when waking up” and “Child seems tired during daytime”. Healthcare professionals requested norm values for age groups due to the wide age span.

Overall, parents and healthcare professionals found the questions relevant and suitable for children in general and for children with ADHD, although some experienced that the questions were less relevant as a child grows older. Questions 9 (“Child sleeps too little”) and 10 (“Child sleeps the right amount”), questions 24 (“Child awakes once during the night”) and 25 (“Child awakes more than once during the night”), and questions 29 (“Child has difficulty getting out of bed in the morning”) and 30 (“Child takes a long time to become alert in the morning”), were perceived to be similar in content and redundant. Question 32 (“Child has appeared very sleepy or fallen asleep during the following: Watching TV”) raised discussions among parents and healthcare professionals. Several screens are frequently used by children in Sweden today, but for various purposes. Watching linear TV broadcasts is quite uncommon, while instead children use tablets, smartphones, computers, or view content from streaming services on the TV. There is also more variation regarding when this screen exposure occurs, which differs from when TV-watching was an evening tradition. The relevance was considered low for question 32 due to this cultural view of TV in Sweden.

“I don’t know many children who have fallen asleep while watching their tablet, but I do know many adults who have fallen asleep while watching TV”. Parent.

The instructions about how to complete the questionnaire and response options were experienced as being clear. The recall period of the previous week was experienced as too short a period to evaluate children’s sleep. Healthcare professionals reasoned that some of the children’s parents may be divorced and that there is a lack of guidelines on how to use the questionnaire in such a case. It was suggested that each parent should complete a questionnaire or that the parents complete it together if the child moved between parents more frequently than every other week.

Healthcare professionals found CSHQ-SWE to be applicable in their work at the clinic. Many of the questions were relevant to their contact with parents regarding their patients, and the questionnaire could be useful for systemizing sleep assessment. They described that it may be used as a basis for conversation, screening, or evaluation in some instances but that the questionnaire was too comprehensive to use on all patients.

#### Content validity ratio and content validity index

CVR was calculated for all 33 items and the four non-scored inquiries. Six of the 37 items were within the CVR range 0.80 to 1.00 (questions 1, 9, 22, 23, (a), (b)), fulfilling criteria for minimum acceptable value (CVR 0.62). Twenty-two of the remaining 31 items were within the CVR range of 0.00 to 0.60 (questions 2, 5, 6, 7, 8, 10, 11, 12, 14, 16, 17, 18, 19, 20, 21, 24, 25, 30, 31, 32, (c), (d)) indicating agreement by half of the panel. Negative CVR values ranging between −0.20 and −0.60 were found for nine items (questions 3, 4, 13, 15, 26, 27, 28, 29, 33). The CVI for all questions was 0.22 (Table 4).

**Table 4:** Lawshe’s CVR for each question, and CVI for all questions in the CSHQ-SWE

Questions	Lawshe’s CVR
9, 22, (b)	1.00
1, 23, (a)	0.80
5, 25	0.60
6, 8, 11, 19, (c), (d)	0.40
2, 10, 12, 16, 18, 20, 31	0.20
7, 14, 17, 21, 24, 30, 32	0.00
3, 4, 15, 26, 27, 29, 33	−0.20
28	−0.40
13	−0.60
**CVI**	0.22

CVI, content validity index; CVR, content validity ratio

#### Revisions

All suggestions, remarks, and thoughts that emerged during the interviews were carefully evaluated by the multidisciplinary team and adjustments were made when consensus was reached. The multidisciplinary team decided that questions 1–33 should remain as in the original CSHQ as this would enable comparisons between countries. However, the opinions of the participants could be of relevance if further adjustments are to be made to the original questionnaire.

The CSHQ-SWE was revised regarding the non-scored inquiries (Table 5). Even if the CVR was above the minimum acceptable value for inquiries (a) and (b), the healthcare professionals requested more information regarding the time when children fall asleep (a2), the division of sleep each day into nighttime sleep (b1), and naps (b2), and the number of night awakenings that usually occur (c2). These revisions are still compatible with other versions of the CSHQ and provide important aspects to clinicians.

**Table 5:** Revisions made to the non-scored inquiries of CSHQ-SWE

Original [3]	CSHQ-SWE*
(a) Write in child’s bedtime: _____	(a1) Write in child’s bedtime: _____
	(a2) Write in time when child fell asleep: _____
(b) Child’s usual amount of sleep each day: _____ hours and _____ minutes (combining nighttime sleep and naps)	(b1) Child’s usual amount of sleep each day for nighttime sleep: _____ hours and _____ minutes
	(b2) Child’s usual amount of sleep each day for naps: _____ hours and _____ minutes
(c) Write the number of minutes a night waking usually lasts: _____	(c1) Write the number of minutes a night waking usually lasts: _____
	(c2) Write the number of night wakings usually occurring during one night: _____

* The English translation of the Swedish revisions is slightly adjusted from that in the paper version of the CSHQ-SWE

## Discussion

The aim of this instrument development study was to translate and culturally adapt the CSHQ to a Swedish context and to assess the validity and reliability of the CSHQ-SWE in children with ADHD. Weak to moderate correlations were found between parent-estimated sleep through CSHQ-SWE and children’s objectively measured sleep through sleep actigraphy. Internal consistency was acceptable for most subscales. The overall results of face and content validity varied, where comprehensiveness, relevance, comprehensibility, and applicability of the CSHQ-SWE were satisfactory overall, but where CVR spanned from low to high and CVI was low. Utilizing both quantitative and qualitative data to validate the CSHQ-SWE strengthens the results. By asking both parents and healthcare professionals, several perspectives could inform the validation process and increase the applicability of CSHQ-SWE in both clinics and research.

### Concurrent validity

The weak to moderate correlations found in the present study between subjectively (through CSHQ-SWE) and objectively (through sleep actigraphy) measured sleep is partly in line with previous findings. Lucas-de la Cruz et al. [9] assessed concurrent validity with selected specific items and found moderate correlations between item 14 and item 17 with objectively measured sleep onset latency by sleep actigraphy, and with item 27 for sleep actigraphy measured awakenings. Duraccio et al. [36] found a weak correlation between the subscale “Daytime Sleepiness” and actigraphy measured minutes asleep. Both Duraccio et al. [36] and Lucas-de la Cruz et al. [9] included children from 4 to 6 and 7 years of age respectively, which is younger than the sample in the present study. Markovich et al. [21] assessed children aged 6–12 and did not find any correlations between CSHQ subscales and objectively measured sleep by polysomnography. It is difficult to draw any conclusions since methodologies differ between the present study and previous studies. The incorporation of sleep actigraphy or polysomnography in previous studies, each with different sleep measurement approaches, adds complexity to interpretation, highlighting the need for further investigation to ensure robust conclusions. Notably, Waldon et al. [26] found significant correlations between polysomnography and sleep actigraphy for various sleep measures, including those utilized in our study (SOL, WASO, TST, and SE), although variability was observed, particularly among children with ADHD not undergoing medication treatment. Even if actigraphy is considered as a valid assessment method to be used in evaluation of sleep patterns in children, Meltzer et al. [25] highlights the importance of establishing standardized recommendations for the use and reporting of actigraphy in pediatric sleep research, addressing issues such as poor specificity in detecting wake after sleep onset and scoring rule inconsistencies, while defining acceptable sensitivity and specificity levels.

The correlations found between CSHQ-SWE total score and sleep actigraphy measured SE, “Sleep Onset Delay” and sleep actigraphy measured SOL, “Night Wakings” and sleep actigraphy measured WASO, and “Total daily sleep duration” and sleep actigraphy measured TST followed a logical structure, which was based on subscale/item names and items included in each subscale that were assessed in the correlation analyses. This provides some support to the concurrent validity between the CSHQ-SWE and actigraphy measured sleep, but more research is needed to determine whether this is only specific for the included population, or if similar results can be found in other populations.

### Reliability

Five of the seven subscales in the present study attained an acceptable level for internal consistency as measured by McDonald’s Omega H. The internal consistency in the present study (Omega H = 0.573–0.827) was in concordance with Cronbach’s α (0.55–0.85) presented by Parreira et al. [19] for a clinical group (ADHD group and sleep problem group). Internal consistency was also in concordance with, or slightly higher than, previous findings from the US (Cronbach’s α = 0.36–0.70) [3], Norway (Cronbach’s α = 0.26–0.79) [16], Portugal (Cronbach’s α = 0.44–0.74) [11], Germany (Cronbach’s α = 0.23–0.70) [37], Italy (Cronbach’s α = 0.550–0.712) [12], and Japan (Cronbach’s α = 0.42–0.68) [10]. McDonald’s Omega H was 0.573 and considered to be below the acceptable level for the subscale “Parasomnias”, which is a finding in line with the study by Hanset et al. [16] where Cronbach’s α was reported to be 0.58. A possible reason for the low internal consistency for the subscale “Parasomnias” may be that the various sleep problems assessed in the questions included in the subscale are not necessarily related to one another. No McDonald’s Omega H could be calculated due to the poor distribution of answers to the questions included in the subscale “Sleep Disordered Breathing”. Parreira et al. [19] found similar results for internal consistency of the subscale “Sleep Disordered Breathing” between the clinical group (ADHD group and sleep problem group) and the non-clinical group, which could suggest that the group of children with ADHD in the present study may not be representative of the population at large in this matter. A larger sample with a broader distribution of answers, would have been needed to increase the confidence of the findings and enable calculation of internal consistency of the “Sleep Disordered Breathing” subscale.

### Face and content validity

Comprehensiveness, relevance, comprehensibility, and applicability of CSHQ-SWE were satisfactory overall according to the parents and healthcare professionals. Issues were, however, raised concerning redundancies, length of questionnaire, and recall period. Issues regarding relevance were also highlighted, both qualitatively in interviews and quantitatively in ratings of content validity ratio. The questions were perceived to be less relevant the older the child becomes. Six items fulfilled criteria for minimum acceptable CVR, 22 items fulfilled agreement by half of the panel, and nine items were not considered to be essential. The low CVI of 0.22 may be the result of the issues raised by parents and healthcare professionals. Gios et al. [7] assessed content validity coefficients and found an overall score for the scale of 0.88 demonstrating acceptable concordance in the expert panel for the Brazilian version of CSHQ. It is unclear why the results differ between the present study and the study by Gios et al. [7], but our panel had a larger sample size and consisted of several parents and healthcare professionals, which may have contributed to a larger variety in relevance scoring. Another explanation could be that the target group in the study by Gios et al. [7] was typically developing children, whereas our target group was children with ADHD.

Interviews with parents and healthcare professionals resulted in minor revisions of the non-scored inquiries in CSHQ-SWE. The decision to adhere to the original CSHQ and continue to include 33 items was supported by previous studies also including all items [8, 9, 11] in order to enable comparisons with other countries and with the original CSHQ [3]. No previous study has to our knowledge presented elaborate results from interviews in validation processes [7, 11], which could be useful in further development of the original CSHQ [3].

### Limitations

One limitation of the present study could be that the face and content validity process did not precede the concurrent validity and reliability processes, as described by Tsang et al. [38]. The CSHQ-SWE was, however, piloted among eight parents before it was carried out, with no major concerns from the parents regarding the comprehensiveness, relevance, and comprehensibility, supporting its distribution. However, content validity for each question rated by the parents and the healthcare professionals showed that CVR spanned from low to high and that CVI was low. This needs to be further investigated. Test-retest reliability, which is a valuable component in a validation process [38], was not performed due to practical and resource constraints. Additionally, repeated questionnaire completion could cause fatigue or reduced compliance among informants.

The sample size was too small to evaluate construct validity of CSHQ-SWE, which is a limitation regarding the psychometric properties. Construct validity is an important aspect of instrument development [38] and should be prioritized in future studies with a larger sample size combining non-clinical and clinical samples. The inclusion of a non-clinical sample in future studies would also enable the analysis of a cut-off suitable for the Swedish context and for the ADHD population, which Parreira et al. [19] demonstrated with a higher cut-off for a Portuguese population than Owens et al. [3] had demonstrated for a US population.

The sample in the present study had uncomplicated ADHD, meaning that findings regarding the psychometric properties of the CSHQ-SWE may not be generalized to other populations with and without medical diagnoses. It’s important to note that our study specifically targeted children with sleep problems rather than those with clinically diagnosed sleep disorders, which may influence the applicability of our results to broader populations. Thus, further studies are needed to determine how and if CSHQ-SWE can be applicable to children in the general population as well as in other populations with medical diagnoses.

### Clinical implications

Mild–severe sleep problems affect 73.3% of children with ADHD [18], highlighting the need for thorough sleep assessment in this population. Validating the CSHQ-SWE for children with ADHD would provide clinicians with a standardized tool to evaluate sleep problems, potentially allowing for more consistent comparisons of sleep interventions, treatments, and medications. Additionally, it could help assess the equity of care related to sleep problems and ADHD across Sweden. The CSHQ-SWE can be a valuable tool for healthcare professionals when taking a sleep history, as it covers various dimensions of sleep, facilitating a more comprehensive dialogue to better understand children’s sleep issues. Given the high prevalence of sleep disorders such as sleep disturbances, restless sleep, and restless legs syndrome in children with ADHD, it is important that these conditions are not overlooked, as the CSHQ does not specifically focus on them.

## Conclusions

CSHQ-SWE demonstrated acceptable concurrent validity with objectively measured sleep and internal consistency, whereas the overall results of face and content validity assessment varied. The instrument needs to be further evaluated regarding construct validity, responsiveness, test-retest reliability, and its generalization to other populations.

### Electronic supplementary material

Below is the link to the electronic supplementary material.

Supplementary Material 1

## Data Availability

The datasets generated and/or analyzed during the current study are not publicly available due to legal/ethical reasons. The CSHQ-SWE is available upon request from Ingrid Larsson (ingrid.larsson@hh.se).

## Abbreviations

ADHD: Attention Deficit Hyperactivity Disorder
BCa: Bias Corrected and Accelerated
CAMHS: Child and Adolescent Mental Health Service
CI: Confidence Interval
CSHQ: Children’s Sleep Habits Questionnaire
CSHQ-SWE: Children’s Sleep Habits Questionnaire - Swedish Version
CVI: Content Validity Index
CVR: Content Validity Ratio
DSM-5: the Diagnostic and Statistical Manual of Mental Disorders, Fifth Edition
IQR: Interquartile Range
Min: Minutes
Q: Quartile
SD: Standard Deviation
SE: Sleep Efficiency
SOL: Sleep Onset Latency
TST: Total Sleep Time
WASO: Wake After Sleep Onset
