# Supplementary material for: Validity and reliability of the Swedish version of the Children’s Sleep Habits Questionnaire (CSHQ-SWE)
Source: BMC Pediatr. 2024 May 31;24:378. doi: 10.1186/s12887-024-04859-z (PMC11140946; doi:10.1186/s12887-024-04859-z)
Supplement: Supplementary file 1 — Supplementary Material 1 [file 12887_2024_4859_MOESM1_ESM.pdf]

Additional file 1 – CSHQ items, subscales, and non-scored inquiries

CSHQ items

1) Child goes to bed at the same time at night
2) Child falls asleep within 20 minutes after going to bed
3) Child falls asleep alone in own bed
4) Child falls asleep in parent's or sibling's bed
5) Child needs parent in the room to fall asleep
6) Child struggles at bedtime (cries, refuses to stay in bed, etc.)
7) Child is afraid of sleeping in the dark
8) Child is afraid of sleep alone
9) Child sleeps too little
10) Child sleeps the right amount
11) Child sleeps about the same amount each day
12) Child wets the bed at night
13) Child talks during sleep
14) Child is restless and moves a lot during sleep
15) Child sleepwalks during the night
16) Child moves to someone else's bed during the night (parent, brother, sister, etc.)
17) Child grinds teeth during sleep (your dentist may have told you this)
18) Child snores loudly
19) Child seems to stop breathing during sleep
20) Child snorts and/or gasps during sleep
21) Child has trouble sleeping away from home (visiting relatives, vacation)
22) Child awakens during night screaming, sweating, and inconsolable
23) Child awakens alarmed by a frightening dream
24) Child awakes once during the night
25) Child awakes more than once during the night
26) Child wakes up by him/herself
27) Child wakes up in negative mood
28) Adults or siblings wake up child
29) Child has difficulty getting out of bed in the morning
30) Child takes a long time to become alert in the morning
31) Child seems tired
32) Child has appeared very sleepy or fallen asleep during the following: Watching TV
33) Child has appeared very sleepy or fallen asleep during the following: Riding in car

CSHQ subscales and items

Subscales	Items
1. Bedtime Resistance	1, 3, 4, 5, 6, 8
2. Sleep Onset Delay	2
3. Sleep Duration	9, 10, 11
4. Sleep Anxiety	5, 7, 8, 21
5. Night Wakings	16, 24, 25
6. Parasomnias	12, 13, 14, 15, 17, 22, 23
7. Sleep Disordered Breathing	18, 19, 20
8. Daytime Sleepiness	26, 27, 28, 29, 30, 31, 32, 33

CSHQ non-scored inquiries

Bedtime
Write in child's bedtime: ___________________

Sleep Behavior
Child's usual amount of sleep each day: ____________ hours and ____________ minutes
(combining nighttime sleep and naps)

Waking During the Night
Write the number of minutes a night waking usually lasts: _________________

Morning Waking/Daytime Sleepiness
Write in the time of day child usually wakes in the morning: _______________

Reference: Owens JA, Spirito A, McGuinn M: **The Children's Sleep Habits Questionnaire (CSHQ): psychometric properties of a survey instrument for school-aged children**. *Sleep* 2000, **23**(8):1043-1051.

Contact: Judith A. Owens, MD, MPH, Director of Sleep Medicine, Boston Children's Hospital, Professor of Neurology, Harvard Medical School, owensleep@gmail.com, Judith.owens@childrens.harvard.edu
